# Fatigue and long duration of infection are associated with worsen motor and non‐motor symptoms in Parkinson's disease following Omicron COVID‐19 pandemic

**DOI:** 10.1002/brb3.3396

**Published:** 2024-02-07

**Authors:** Wenbiao Xian, Lishan Lin, Wanling Wu, Fengjuan Su, Zhong Pei

**Affiliations:** ^1^ Department of Neurology The First Affiliated Hospital, Sun Yat‐sen University Guangzhou China; ^2^ Guangdong Provincial Key Laboratory of Diagnosis and Treatment of Major Neurological Diseases National Key Clinical Department and Key Discipline of Neurology Guangzhou China

**Keywords:** COVID‐19, motor symptom, non‐motor symptom, Parkinson's disease

## Abstract

**Background:**

Coronavirus disease 2019 (COVID‐19) may influence the clinical course and symptoms of chronic neurological diseases, such as Parkinson's disease (PD), which can persist even after recovery from the infection. This longitudinal study aimed at exploring the impact of the COVID‐19 on motor and non‐motor symptoms and the related risk factors for exacerbation of PD symptoms.

**Methods:**

One hundred and two PD patients underwent a first assessment between September 2022 and November 2022 (*T*0) before Omicron COVID‐19 pandemic. They were then contacted again and asked to complete the second assessment between December 2022 and February 2023 (*T*1) following Omicron infection. Movement Disorders Society Unified PD Rating Scale Part III, Non‐Motor Symptoms Scale, Fatigue Severity Scale (FSS), and quality of life were investigated.

**Results:**

Ninety‐five PD patients (93.1%) with COVID‐19 for the first time were mild cases. However, 55 patients (55.9%) experienced worsening motor symptoms of PD after recovering from the infectious symptoms. Preinfection FSS score (odds ratios [OR] 2.062, 95% confidence interval [CI] 1.081–3.933, *p* = .028) and duration of infection (OR 1.232, 95% CI 1.024–1.481, *p* = .027) were independent risk factors for the worsening of motor symptoms. PD patients with post‐COVID‐19 fatigue were more likely to experience worsened non‐motor symptoms, resulting in an impaired quality of life.

**Conclusion:**

This study confirms the impact of the Omicron COVID‐19 pandemic on the motor and non‐motor symptoms of PD, suggesting that management of related factors, including fatigue and duration of infection, may be beneficial in preventing or dealing with the exacerbation of PD symptoms after infection.

## Graphical abstract

Preinfection Fatigue Severity Scale (FSS) score and duration of infection are risk factors for the worsening of motor symptoms of Parkinson's disease following Omicron coronavirus disease 2019 (COVID‐19) pandemic. Patients with higher FSS scores and longer duration of infection were more likely to experience worsening motor symptoms after recovery from infectious symptoms of COVID‐19.

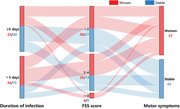

## 1 INTRODUCTION

The coronavirus disease 2019 (COVID‐19) pandemic caused by the severe acute respiratory syndrome coronavirus 2 (SARS‐CoV‐2) has greatly impacted on central nervous system (CNS). A substantial number of COVID‐19 patients developed neurological manifestations, including fatigue, headache, dizziness, syncope, and seizures (Asadi‐Pooya & Simani, 2020). In addition, COVID‐19 also influenced the clinical course and symptoms of chronic neurological diseases, such as Parkinson's disease (PD) (Cartella et al., 2021). The current literature shows that PD patients who contracted COVID‐19 had a decline in motor functions and non‐motor symptoms (El‐Qushayri et al., 2022). A cohort study revealed that COVID‐19 caused a significant deterioration in motor performance, motor fluctuations, and non‐motor symptoms of PD, requiring therapy adjustment in one third of cases. The clinical deterioration was explained by the infection‐related mechanisms and impaired pharmacokinetics of dopaminergic therapy (Cilia et al., 2020). Another retrospective study also reported a worsening of motor and non‐motor symptoms with a significant increase in disease progression during the COVID‐19 pandemic (Shalash et al., 2022).

Infections are a common cause of exacerbations of PD symptoms (Brugger et al., 2015). COVID‐19 infection may have a direct detrimental effect on PD motor and non‐motor symptoms through direct or secondary CNS involvement. In addition, indirect effects such as social isolation, pharmacodynamic effects, dramatic changes in routine, the impact of stress and anxiety as well as prolonged immobility are all likely to have negative effects on motor and non‐motor symptoms and quality of life in PD (Helmich & Bloem, 2020).

Previous studies have reported that COVID‐19 not only aggravates motor and non‐motor symptoms (Brown et al., 2020; D'Iorio et al., 2022) but also exacerbates the disease progression in PD patients (Shalash et al., 2022). Many of these studies have focused on the relationship between COVID‐19 pandemic and clinical features of PD. However, which people with PD are more likely to experience worsening symptoms after COVID‐19 remains poorly studied. China witnessed a surge of Omicron variant infections after the policy adjustment on December 7, 2022, yielding an estimated infection rate of 87.54% in the population (Bai et al., 2023). During the epidemic, a large number of patients with PD experienced COVID‐19, which provides a unique opportunity to address pressing questions with respect to the mechanisms that contribute to the deteriorates of PD symptoms after SARS‐CoV‐2 infection.

In this study, we explore the impact of the COVID‐19 on patients with PD during the Omicron variant outbreak in mainland China between December 2022 and January 2023. This longitudinal study aimed at exploring the changes of clinical symptoms in patients with PD following COVID‐19 pandemic, in comparison with the pre‐pandemic status, thereby potentially helping answering unresolved questions regarding the risk factors contribute to the deteriorates of PD symptoms following Omicron variant infection.

## 2 METHODS

### 2.1 Participants

Participants were recruited from Guangzhou Precision Medicine Parkinson's Cohort (GPMP).

Participants in this cohort are regularly followed up by the Movement Disorder Clinic of the First Affiliated Hospital of Sun Yat‐sen University (Guangzhou, China). Participants had a diagnosis of PD verified by a specialty neurologist according to the International Parkinson and Movement Disorders Society (MDS) diagnostic criteria (Postuma et al., 2015). The inclusion criteria for this study were as follows: (a) diagnosis of COVID‐19 was confirmed by a positive test for SARS‐CoV‐2 using a virologic test (i.e., nucleic acid amplification test or antigen test); (b) recovery from infectious symptoms of COVID‐19 for at least 14 days and within 60 days from the infection (*T*1 follow‐up); and (c) participants had been visited by a neurologist within a 3‐month period before infection (*T*0). Patients excluded include patients with acquired or atypical parkinsonism, those who did not complete the follow‐up assessment, those who were assessed with a time interval of more than 3 months apart from *T*1 to *T*0, and those who underwent brain surgery (before or during the follow‐up period). The research was conducted after PD patients provided their consent approved by the Local Ethics Committee.

### 2.2 Data collection

The participants were recruited from GPMP and their motor symptoms and non‐motor symptoms of PD patients were well recorded. Participants underwent the first assessment between September 2022 and November 2022 (*T*0), before the COVID‐19 outbreak. After the onset of COVID‐19, they were contacted again and asked to complete the second clinical assessment between December 2022 and February 2023 (*T*1). The time interval between *T*1 and *T*0 was required to be less than 3 months to minimize the impact of PD progression on changes in clinical features and mitigate the potential biases. The severity of COVID‐19 was determined as per the following criteria (Wei, 2020): (1) mild: The clinical symptoms were mild, and there was no evidence of pneumonia on imaging; (2) moderate: evidence of lower respiratory disease during clinical assessment or imaging with oxygen saturation (SpO2) ≥93%; (3) severe: either respiratory distress, respiratory rate  > 30/min, SpO2  < 93% at rest, or arterial oxygen tension/fraction of inspired oxygen (PaO2/FiO2)  <300 mm Hg; (4) critical: either respiratory failure requiring ventilation, hemodynamic instability, other organ damage or intensive care unit admission. Motor symptoms were assessed using the Movement Disorders Society Unified PD Rating Scale Part III (MDS‐UPDRS‐III) and Hoehn and Yahr (H&Y) stage for disease severity. Non‐motor symptoms were assessed using the Non‐Motor Symptoms Scale (NMSS). Cognition was evaluated by the Mini–Mental State Examination (MMSE) and Montreal Cognitive Assessment (MoCA). The Parkinson Disease Quality of Life Questionnaire‐8 (PDQ‐8) was used to measure health‐related quality of life. The PDQ‐8 summary index (PDQ‐8‐SI) is summed over the eight dimensions and standardized from 0 to 100. Fatigue was measured by the Fatigue Severity Scale (FSS), which is the most commonly used scale to assess the conditions associated with fatigue. It was the only scale among those evaluated to receive the “recommended” classification of the MDS task force for both screening and grading the severity of fatigue (Friedman et al., 2010). Subjects who scoring >4 on the FSS were classified as fatigued (Zuo et al., 2016). The dosages of dopaminergic medications were converted to daily levodopa‐equivalent daily dose (Tomlinson et al., 2010). All assessments were performed during the medication‐on (med‐on) phase, as patients' symptoms may worsen after infection with COVID‐19 and not cooperate with the assessment. Whether the patient's motor symptoms worsened or not was also evaluated comprehensively by two neurologists based on the patient's presentation, changes in MDS‐UPDRS‐III scores, and increase of daily dose of dopaminergic medication.

### 2.3 Statistical analysis

All statistical analyses were performed using IBM SPSS software package version 18.0 (IBM Corp.). Descriptive statistics were provided for continuous (medians and interquartile ranges or means ± standard deviations) and categorical (count and percentage) variables. The paired Wilcoxon signed‐rank test was used to compare the clinical data between baseline (*T*0) and follow‐up (*T*1). Binary logistic regression was performed to identify potential factors associated with worsening of motor symptoms of PD after Omicron infection and odds ratios (OR) along with 95% confidence intervals (CI) were calculated. The significance was set at *p* < .05.

## 3 RESULTS

### 3.1 Symptoms of COVID‐19 in PD patients

A total of 135 PD patients were included in the study. Thirty‐three out of 135 participants did not give their consent for lack of interest or the clinical data was incomplete in *T*0, so the final sample was of 102 PD patients. The sample was composed of 57 males (55.9%) and 45 females (44.1%), with a mean age of 62.7 ± 9.7 years (range 29–82), disease duration of 7.0 ± 4.7 years (range 1–25), and H&Y stage of 2.3 ± 0.8 (range 1–4). The time between the diagnosis of COVID‐19 and the neuropsychological assessment was an average of 36.8 ± 10.3 days (range 20–56). All patients were experiencing COVID‐19 for the first time. COVID‐19 symptoms were mild in 95 cases (93.1%); 5 cases (4.7%) had moderate illness and showed evidence of lower respiratory disease during clinical assessment; only 2 patients were hospitalized (1.9%) as a result of pneumonia with hypoxemia. No patient required mechanical ventilation or died. The mean duration of the COVID‐19 symptoms was 5.4 ± 2.7 days (range 1–14). Motor and non‐motor symptoms of all participants at baseline (*T*0) and after Omicron infection (*T*1) are reported in Table 1.

**TABLE 1:** Motor and non‐motor symptoms at baseline and *T*1 follow‐up.

Variable	*T*0	*T*1	*Z*	*p*
MDS‐UPDRS‐III (med‐on)	30.6 ± 12.6	31.5 ± 13.1	−2.639	.008
LEDD, mg	509.2 ± 288.4	539.4 ± 294.1	−5.771	<.001
FSS score	2.2 ± 0.9	3.7 ± 1.6	−7.771	<.001
PDQ‐8‐SI	12.1 ± 7.8	14.2 ± 9.1	−3.234	.001
MMSE	28.1 ± 2.1	28.0 ± 2.3	−.929	.353
MoCA	25.0 ± 4.2	24.5 ± 4.1	−1.690	.091
NMSS total score	28.6 ± 19.4	34.9 ± 25.3	−6.106	<.001
Cardiovascular	0.4 ± 1.6	0.5 ± 1.8	−1.841	.066
Sleep/Fatigue	3.7 ± 3.5	5.5 ± 5.3	−4.792	<.001
Mood/Cognition	2.9 ± 6.1	4.4 ± 8.3	−3.829	<.001
Perceptual problems/Hallucinations	0.5 ± 1.6	0.8 ± 2.3	−2.371	.018
Attention/Memory	2.1 ± 2.6	2.9 ± 3.4	−2.858	.004
Gastrointestinal	3.6 ± 3.9	3.7 ± 3.9	−.532	.595
Urinary	4.1 ± 4.4	4.6 ± 4.7	−2.458	.014
Sexual function	8.5 ± 5.8	8.4 ± 5.8	−1.282	.200
Miscellaneous	2.8 ± 3.4	4.1 ± 4.6	−4.823	<.001

*Note*: The values are expressed as mean ± standard deviation.

Abbreviations: FSS, Fatigue Severity Scale; H&Y, Hoehn and Yahr; LEDD, levodopa equivalent daily dose; MDS‐UPDRS‐III, Movement Disorders Society Unified PD Rating Scale Part III; MMSE, Mini–Mental State Examination; MoCA, Montreal Cognitive Assessment; NMSS, Non‐Motor Symptoms Scale; PDQ‐8‐SI, Parkinson's Disease Quality of Life Questionnaire‐8 summary index; T0, before Omicron COVID‐19 pandemic; T1, after Omicron COVID‐19 pandemic.

### 3.2 Worsening of motor symptoms

**FIGURE 1:**
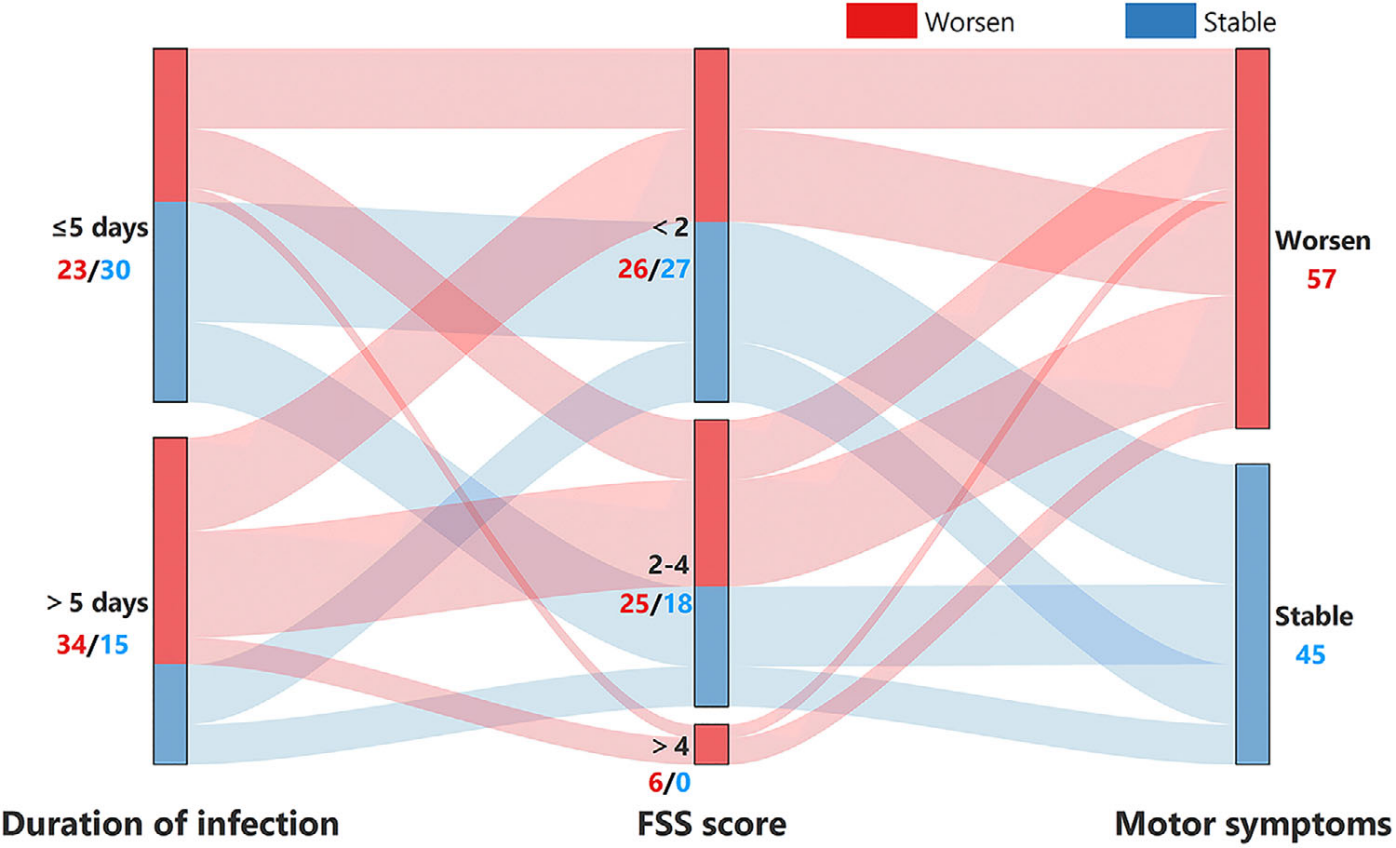
Risk factors for the worsening of motor symptoms following Omicron coronavirus disease 2019 (COVID‐19) pandemic. Parkinson's disease (PD) patients with higher Fatigue Severity Scale (FSS) scores and longer duration of infection were more likely to experience worsening motor symptoms.

There was a significant worsening of MDS‐UPDRS‐III (med‐on) score in patients after Omicron infection (31.5 ± 13.1) compared to baseline (30.6 ± 12.6, *p* < .01). Fifty‐seven patients (55.9%) reported a worsening of motor symptoms after infection. Binary logistic regression analysis was used to evaluate the effects of age, sex, PD disease duration, duration of infection, severity of infection, H&Y stage, MDS‐UPDRS‐III (med‐on), FSS score, and NMSS score on the deterioration of motor symptoms. The results suggested that preinfection FSS score (OR 2.062, 95% CI 1.081–3.933, *p* = .028) and duration of infection (OR 1.232, 95% CI 1.024–1.481, *p* = .027) were independent risk factors for the worsening of motor symptoms after recovery from infectious symptoms of COVID‐19 in PD patients. Specifically, those with higher FSS scores before infection and longer duration of infection were more likely to experience worsening motor symptoms after recovery from infectious symptoms of COVID‐19 (Figure 1). The dopaminergic therapy dosing was significantly increased following Omicron COVID‐19 pandemic (Table 1). After the onset of COVID‐19, 20 patients (19.6%) increased their levodopa dosage, 5 patients (4.9%) increased the dosage of catechol‐O‐methyltransferase inhibitors, 3 patients (2.9%) increased the dosage of dopamine receptor agonists, and 11 patients (10.8%) received rehabilitation.

### 3.3 Changes in fatigue

**FIGURE 2:**
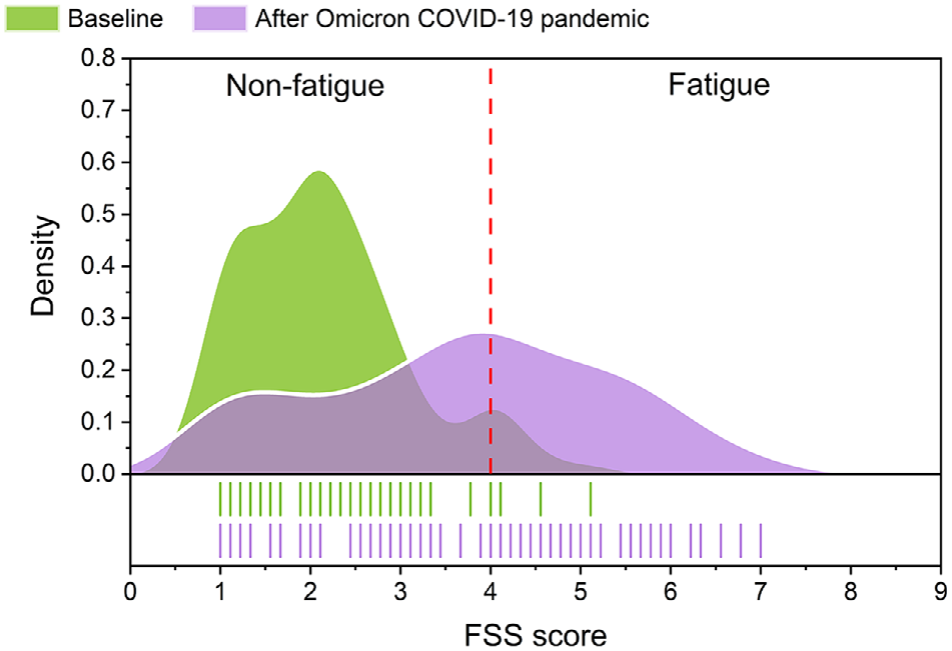
Parkinson's disease (PD) patients presented with fatigue significantly increased following Omicron coronavirus disease 2019 (COVID‐19) pandemic.

At baseline, 6 out of 102 PD patients (5.9%) experienced fatigue (FSS score >4), with an average FSS score of 2.2 ± 0.9. After SARS‐CoV‐2 infection, PD patients exhibited significantly higher FSS scores (3.7 ± 1.6) when compared to the baseline (*p* < .01). There were 42 PD patients (41.2%) experienced fatigue within 14–60 days of recovery from infectious symptoms of COVID‐19 (Figure 2). Post‐COVID‐19 participants with average FSS scores of >4 points and ≤4 points were classified into the fatigue and non‐fatigue groups, respectively.

### 3.4 Worsening of non‐motor symptoms

**FIGURE 3:**
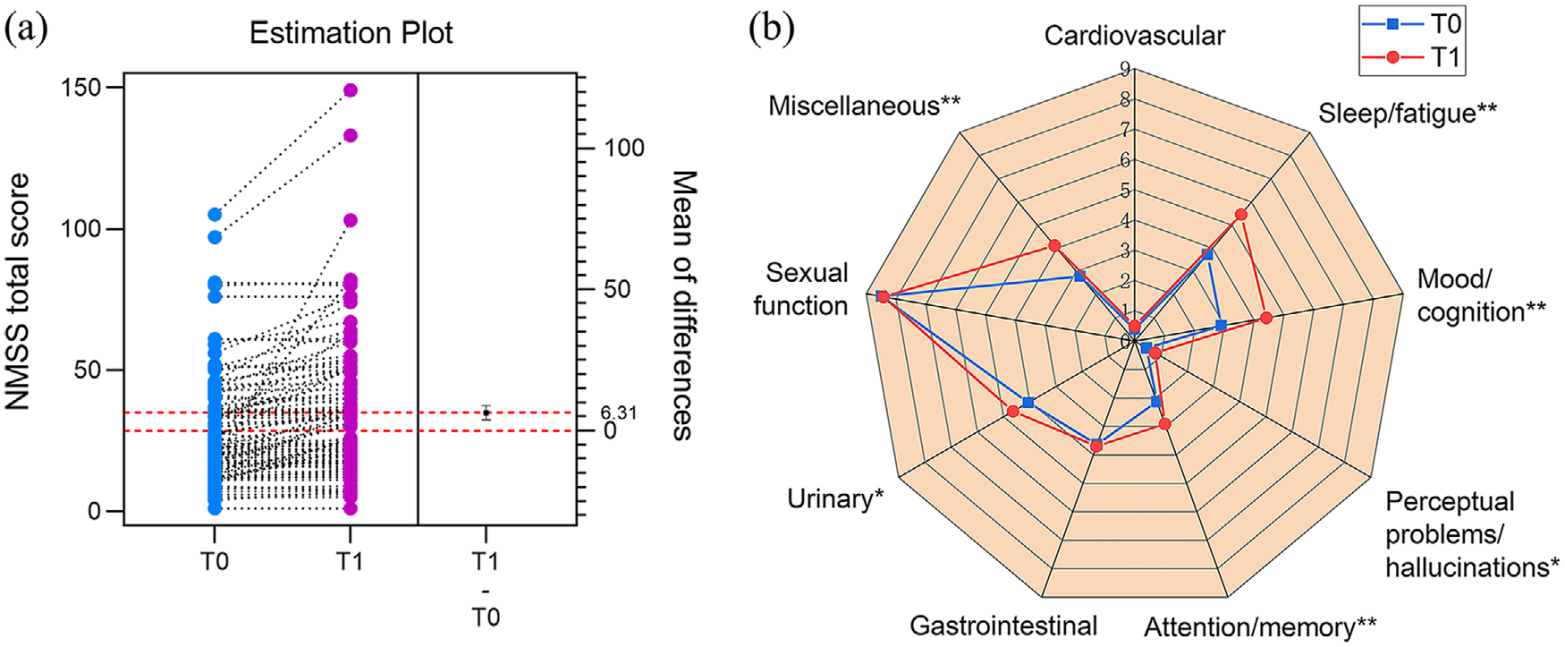
Changes in non‐motor symptoms following Omicron coronavirus disease 2019 (COVID‐19) pandemic: (a) Non‐Motor Symptoms Scale (NMSS) total score significantly worsened from baseline to follow‐up; (b) sleep/fatigue, mood/cognition, perceptual problems/hallucinations, attention/memory, urinary and miscellaneous symptoms were the most significantly affected non‐motor symptoms. *T*0, before Omicron COVID‐19 pandemic; *T*1, after Omicron COVID‐19 pandemic; *p < .05, **p < .01.

There was no significant difference in MMSE and MoCA scores between *T*0 and *T*1; however, the NMSS total score significantly worsened from baseline to follow‐up (Figure 3a), with an average change score of 6.3 ± 12.9. Post hoc exploratory analyses of NMSS domains showed significant worsening scores in sleep/fatigue, mood/cognition, perceptual problems/hallucinations, attention/memory, urinary, and miscellaneous domains at *T*1 follow‐up (Figure 3b). After recovery from infectious symptoms of COVID‐19, NMSS total score worsened more significantly in PD patents in fatigue group (42.9 ± 28.8) than non‐fatigue group (24.9 ± 15.2, *p* < .001).

### 3.5 Worsening of quality of life

**FIGURE 4:**
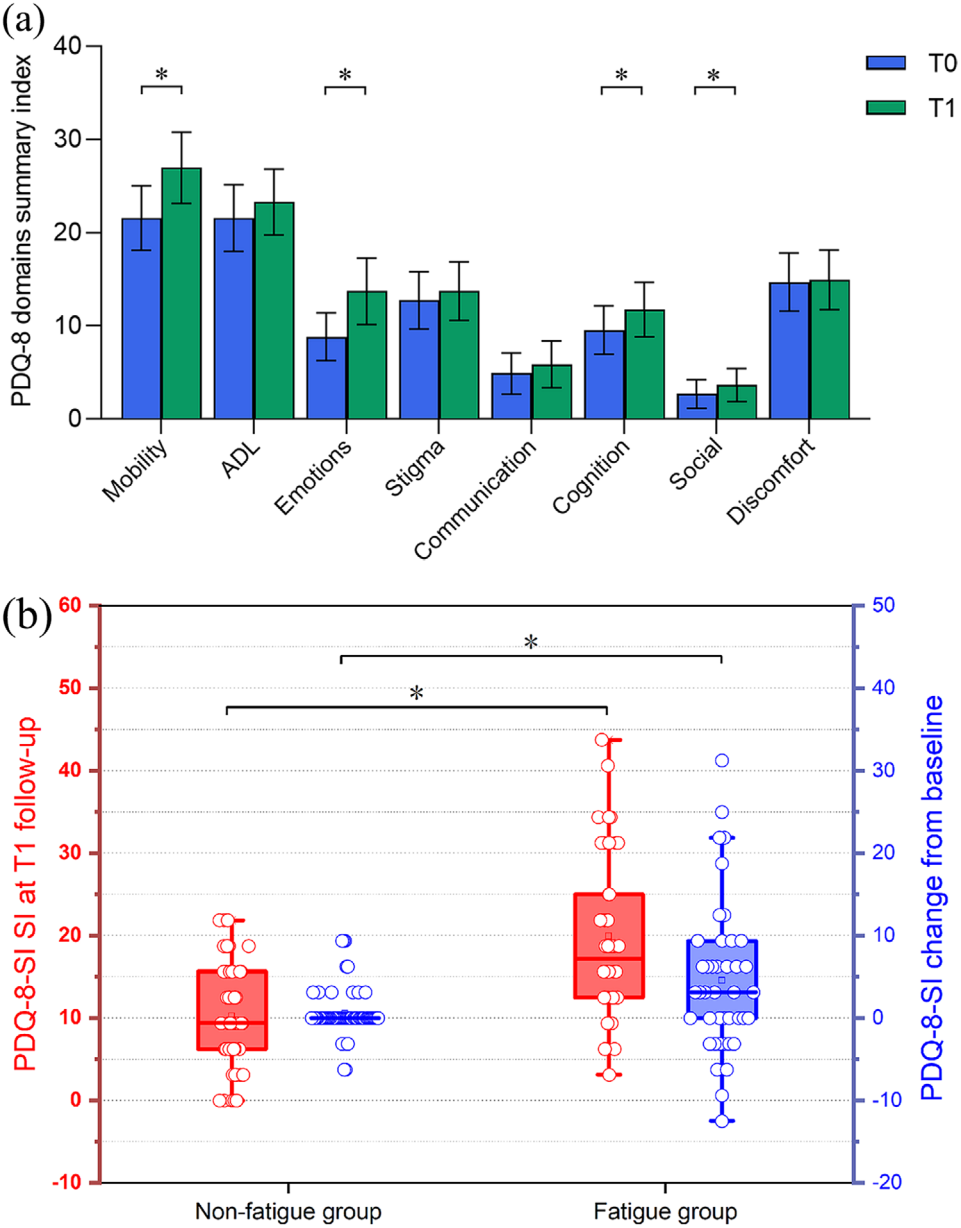
Worsening of quality of life following Omicron coronavirus disease 2019 (COVID‐19) pandemic: (a) Parkinson's Disease Quality of Life Questionnaire‐8 summary index (PDQ‐8‐SI) domains/items at baseline (blue) and *T*1 follow‐up (green) with 95% confidence intervals (error bars); (b) patents in fatigue group exhibited a worse PDQ‐8‐SI score than non‐fatigue group following Omicron infection. *T*0, before Omicron COVID‐19 pandemic; *T*1, after Omicron COVID‐19 pandemic; *p < .05.

PD patients after recovery from infectious symptoms of COVID‐19 showed a significant worsening PDQ‐8‐SI compared to the baseline. PDQ‐8 domain analyses revealed that mobility, emotions, cognition, and social domains were significantly worsened after infection. PD patients in the fatigue group exhibited a worse PDQ‐8‐SI (19.9 ± 9.6) than those in the non‐fatigue group (10.3 ± 6.2) following Omicron COVID‐19 pandemic (*p* < .001) (Figure 4).

## 4 DISCUSSION

The aim of the present study was to investigate the impact of Omicron COVID‐19 pandemic on the motor and non‐motor symptoms of PD and the related risk factors for exacerbation of PD symptoms. Overall, our results showed that 55 out of 102 PD patients (55.9%) experienced worsening motor symptoms after the first Omicron infection, with an increase of daily dose of dopaminergic medication. Preinfection FSS score and duration of infection were identified as independent risk factors for the worsening of motor symptoms after recovery from infectious symptoms of COVID‐19. Remarkably, FSS and NMSS scores deteriorated significantly after infection, which greatly affected patients’ quality of life (with worse PDQ‐8‐SI).

In our study, 95 PD patients (93.1%) with Omicron infection were mild cases, suggesting that Omicron variant was less severe than the previous circulating variants. However, PD patients with mild COVID‐19 have been reported to experience substantial worsening of motor and non‐motor symptoms. The current findings are consistent with those of previous studies (Anghelescu et al., 2022; Mai et al., 2022; Wolff et al., 2022). The relatively mild severity COVID‐19 in this cohort may be due to Omicron variant, which is currently the dominant variant of coronavirus worldwide and can cause mild or even no symptoms for many people (Zhang et al., 2022). As the infection symptoms are generally mild in our cohort, it is undeniable that the changes in social conditions (e.g., less frequent outings, less exercise) due to the COVID‐19 epidemic may potentially worsen the motor and non‐motor symptoms of PD. Nowadays, increasing attention is directed toward whether SARS‐CoV‐2 infection will cause post‐sequelae in patients with chronic neurological diseases (Meshkat et al., 2020; Sakibuzzaman et al., 2022). According to the recent reports, individuals with neurodegenerative diseases, including Alzheimer's disease and PD, have been negatively impacted by the COVID‐19 pandemic (Barbieri et al., 2022; Fu et al., 2022).

In our study, infection duration and fatigue are significant risk factors for exacerbating motor symptoms in PD patients after recovery from infectious symptoms of COVID‐19. To our knowledge, this is the first study exploring the risk factors affecting the exacerbation of PD symptoms after COVID‐19. Indeed, COVID‐19 can induce a significant worsening of motor performance and motor‐related disability (Cilia et al., 2020). Worsening of motor symptoms was so pronounced in 55.9% of our cases that it prompted neurologists to increase dopaminergic therapy. The aggravation of motor symptoms may be caused either by direct action of SARS‐CoV‐2 or its acute systemic inflammatory response, which eventually alters pharmacokinetics. Indeed, SARS‐CoV‐2 enters the host through cellular receptor angiotensin‐converting enzyme 2, which is highly expressed in human airway epithelia and also dopaminergic neurons (Rodriguez‐Perez et al., 2020). Alpha‐synuclein has been reported to be upregulated after immune stimulation. One of normal function of alpha‐synuclein is to restrict the replication of RNA viruses and protect against virus‐induced neuronal injury (Ait et al., 2020). However, excessive expression of alpha‐synuclein also negatively affects PD pathogenesis and progression. It has been reported that several SARS‐CoV‐2 patients exhibit parkinsonism with abnormal dopamine transporter imaging (Ali et al., 2022; Bouali‐Benazzouz & Benazzouz, 2021). In this regard, we hypothesize that timely use of anti‐COVID‐19 drugs in PD patients (especial those with high preinfection FSS scores) at the early stage of COVID‐19 may shorten the duration of the infection and further prevent the aggravation of motor symptoms.

COVID‐19 can also significantly aggravate a number of non‐motor symptoms. Compared with the baseline, NMSS total score become significantly worsening in PD patients after recovery from infectious symptoms of COVID‐19 in our study. Consistent with previous reports (Mameli et al., 2022), sleep/fatigue, mood/cognition, perceptual problems/hallucinations, attention/memory, and urinary were the most significantly affected non‐motor symptoms in our patients. It should be noted that there are substantial overlapping symptoms between COVID‐19 infection and PD. For example, the most common symptoms of long COVID, including fatigue, cognitive disturbances (loss of concentration and memory deficits), and sleep disturbances, are also the presentation of non‐motor symptoms of PD, which could worsen after onset of COVID‐19 (Leta et al., 2021). Remarkably, FSS scores were markedly worsened in our patients after COVID‐19 onset. COVID‐19 patients with PD who experience fatigue were more likely to have worsening non‐motor symptoms, with worse NMSS scores, leading to impaired quality of life. Fatigue is one of the most common and most disabling symptoms of post‐COVID‐19 syndrome in the population (Ceban et al., 2022). Prevalence rates of fatigue persisting for months after COVID‐19 onset range from 9% to 58% depending on time of follow‐up, study population, and different evaluation methodologies (Augustin et al., 2021; Lopez‐Leon et al., 2021; Stavem et al., 2021). On the other hand, fatigue is also one of the most common non‐motor symptoms in PD and may affect a wide range of everyday activities, cause disability, and reduce quality of life. It can occur at every stage of PD, often persist, and may worsen over time. A meta‐analysis showed that the symptom of fatigue due to COVID‐19 was 1.20 times greater in patients with PD than ones without PD (Afraie et al., 2023).

Fatigue plays an important role in the pathogenesis of both COVID‐19 and PD and is closely related to systemic inflammation. Breton et al. (2021) reported marked increases in the numbers of CD4 + T cells expressing the inflammatory cytokines interleukin (IL)‐2, interferon‐γ, and tumor necrosis factor (TNF)‐α in post‐COVID individuals. Elevated IL‐6 levels were founded in post‐COVID individuals (Fortini et al., 2021; Sonnweber et al., 2021). Similar results are found in PD studies, owing to the conformational modification of abnormal α‐synuclein aggregation, there is a release of a plethora of proinflammatory cytokines through the activation of toll‐like receptor 4 on microglia, subsequently inducing fatigue symptoms (Wang et al., 2021). Inflammatory marker levels, such as IL‐6, soluble IL‐2 receptor, and TNF‐α, are closely associated with fatigue in PD patients (Wang et al., 2021). To date, there is a lack of current evidence‐based therapies for fatigue of PD or post‐COVID‐19 fatigue. Amantadine has complex activity, including antiviral, immunomodulatory, and dopaminergic effects (Chober et al., 2022). It has been used to treat fatigue in other neurological diseases with mixed results (Nourbakhsh et al., 2021; Perez et al., 2020). Recent studies have shown that amantadine is effective in the treatment of COVID‐19 symptoms (Chober et al., 2022; Kamel et al., 2021), suggesting that it may have a potential effect on post‐COVID‐19 fatigue in PD patients (Muller et al., 2023). Rehabilitation is an effective, safe, and well‐tolerated intervention for the recovery of post‐COVID‐19 fatigue (Araujo et al., 2023; Jimeno‐Almazan et al., 2022; Ostrowska et al., 2023).

We acknowledge that our study has some limitations. First, we did not compare the participants with a group of PD patients without COVID‐19, as approximately 87.54% of the population experienced Omicron infection during this period. In our study, the time interval between *T*1 and *T*0 was less than 3 months in order to minimize the impact of PD progression on clinical characteristics changes. Second, most PD patients (93.1%) with COVID‐19 were mild cases caused by Omicron infection, which may not be consistent with previous reports in which patients were infected by other variants. China adhered to the policy of “zero COVID” for nearly 3 years and took active restrictive measures. By December 7, 2022, China decided to radically change the course, and Omicron varieties were widely circulated throughout the country. Consequently, the mild cases due to Omicron infection were dramatically increased. Third, given that patients’ symptoms may become worsening after infection with COVID‐19 and may not cooperate with the assessment, MDS‐UPDRS‐III was evaluated at the med‐on state, which may not fully reflect the severity of the motor symptoms. Nevertheless, we believe that our results are clinically relevant for the understanding of risk factors contribute to the deteriorates of PD symptoms following SARS‐CoV‐2 infection.

In conclusion, this study confirms the impact of the Omicron COVID‐19 pandemic on the motor and non‐motor symptoms of PD during mild COVID‐19, implying the significance of managing related factors, including fatigue and duration of infection, and considering this effect in the adjustment of the treatment strategy to prevent or deal with the exacerbation of PD symptoms after infection. In addition, post‐COVID‐19 fatigue is an important factor for the deterioration of non‐motor symptoms after the COVID‐19 epidemic, indicating that other factors such as inflammation may be involved in the deterioration of PD symptoms. Future longitudinal studies comparing with PD patients without COVID‐19 are needed to identify contributing factors to the worsening of PD symptoms after COVID‐19 onset.

## AUTHOR CONTRIBUTIONS

**Wenbiao Xian**: Writing—original draft; conceptualization; data curation; investigation; methodology; project administration; formal analysis. **Lishan Lin**: Data curation; investigation; validation; methodology; formal analysis; project administration. **Wanling Wu**: Data curation; investigation. **Fengjuan Su**: Conceptualization; writing—review and editing; data curation; validation; supervision. **Zhong Pei**: Conceptualization; writing—review and editing; supervision; funding acquisition.

## CONFLICT OF INTEREST STATEMENT

The authors declare no conflicts of interest.

### PEER REVIEW

The peer review history for this article is available at https://publons.com/publon/10.1002/brb3.3396.

## Data Availability

The data underlying this article will be shared on reasonable request to the corresponding author.
